# *Rhus verniciflua* leaf extract suppresses obesity in high-fat diet-induced obese mice

**DOI:** 10.29219/fnr.v63.3601

**Published:** 2019-09-11

**Authors:** Kohei Suruga, Tsuyoshi Tomita, Kazunari Kadokura, Toshiro Arai

**Affiliations:** 1 Food Function R&D Division, International Operation Department, Kibun Foods Inc., Tokyo, Japan; 2 Laboratory of Veterinary Biochemistry, School of Veterinary Medicine, Nippon Veterinary and Life Science University, Tokyo, Japan

**Keywords:** plant extract, anti-obesity, quercetin derivative

## Abstract

**Background:**

Obesity is a serious health problem in the world. We thought that the activity and safety of natural plants and/or foods are very important in the management of therapy for obesity. *Rhus verniciflua* (*R. verniciflua*) is also known as lacquer tree in Japan and Korea, and it is consumed as food ingredients and/or traditional herbal medicine. We prepared an extract from *R. verniciflua* leaves (Rv-PEM01-99) to develop a new functional food material and/or nutritional supplements.

**Objective:**

This study evaluated the anti-obesity effects of the Rv-PEM01-99 in high-fat diet (HFD)-induced obese mice.

**Design:**

Six-week-old male C57BL/6J mice were divided into three groups: group I (HFD group), group II (HFD + 1% Rv-PEM01-99 group), and group III (HFD + 2% Rv-PEM01-99 group). Throughout the 56-day treatment period, body weights of these mice were checked twice a week. After 56 days, blood biochemical analyses were performed.

**Results:**

In animal studies, no death or abnormalities in food consumption were observed between groups I, II, and III. Body weight gain in the groups administered Rv-PEM01-99 was less than that in group I. In particular, body weight gain in group III was significantly less than that in group I after 52 days of Rv-PEM01-99 administration. In addition, intra-abdominal fat and leptin levels in group III were significantly lower than those in group I. HPLC and LC/MS analysis showed a quercetin derivative as an active compound in Rv-PEM01-99.

**Conclusion:**

Rv-PEM01-99, containing a quercetin derivative, showed anti-obesity effect in HFD-fed mice. It could therefore be useful as food material and/or nutritional supplement for management of obesity.

## Popular scientific summary

- This is the first report about anti-obesity effect of Rv-PEM01-99 from *Rhus verniciflua* leaves on HFD-fed mice.
- Rv-PEM01-99 showed the anti-obesity effect on HFD-fed mice.
- One of the active compounds of Rv-PEM01-99 is a quercitrin, which is a quercetin derivative.
- Rv-PEM01-99 can be used as an edible component and/or nutritional supplement for management of obesity.

Obesity is a serious health problem significantly contributing to reduction in quality of life and lifespan globally (1). World Health Organization (WHO) defines obesity as abnormal or excessive fat accumulation that presents a health risk (2). High body weight and obesity can lead to many serious diseases, including high blood pressure, diabetes, and cancer (3). Therefore, high body weight and obesity are global health challenges, particularly in light of several chronic diseases (4). The main reasons for occurrence of obesity are the delicious and high-energy foods rich in fat (5). In general, controlling obesity requires maintaining an optimal body weight by calorie restriction, sugar restriction, and exercise programs (6). However, following this regimen can be difficult for many obese patients.

Numerous phytochemicals from fruits, vegetables, and herbs have antioxidant activity, which is of considerable practical importance, as they protect the human body from damages induced by reactive oxygen species (ROS) and free radicals (7, 8). Oxidative stress by ROS, including the superoxide anion radicals, hydroxyl radicals, and H_2_O_2_, is linked to the induction of various serious diseases, such as neurodegenerative disorders, cancer, cardiovascular diseases, atherosclerosis cataracts, and inflammation (9). Epidemiological studies have indicated the association of the inactivation of ROS through intake of foods rich in antioxidants, such as fruits, vegetable, and certain cereals, with disease prevention. Furthermore, antioxidant compounds and foods are being widely consumed globally for health reasons (10). Recently, it has been reported that natural herbs and/or natural plants, which have an antioxidant activity, may be an effective treatment option for obesity (11). The potency and safety of these natural plants for long-term treatment are very important in the management of obesity.

We investigated the antioxidant, alpha-glucosidase inhibitory activity, and anti-obesity effects of fruits, vegetables, and herbs. *R. verniciflua*, a member of the Anacardiaceae family, is commonly known as the lacquer tree, which contained urushiols (12). The bark, branch, and stem of *R. verniciflua* are consumed as food ingredients and/or traditional herbal medicine in Korea, and many reports have indicated their antimicrobial (13), anti-inflammatory (14), and cytotoxic properties (15). Kim et al. investigated the antimicrobial and alpha-glucosidase inhibitory effects of *R. verniciflua* heartwood and stem extracts, and showed that the active compounds of these parts were fustin, gallic acid, 3’,4’,7-trihydroxyflavone, and fisetin (13). *R. verniciflua* leaves are consumed as pickles for human health in specific areas of Korea; however, the consumption of these leaves is not widespread. Moreover, Kim et al. reported the protective effect of 70% methanol extract of *R. verniciflua* leaves on human dopaminergic cells (16). However, only a few studies have explored the anti-obesity effect of *R. verniciflua* leaves extract. To develop a new functional food material and/or nutritional supplement, we prepared the plant extract mixture, Rv-PEM01-99 from *R. verniciflua* leaves. Previously, we studied the antitumor and antiapoptotic effects (17, 18) of *R. verniciflua* leaf extract in tumor-bearing dogs (19), and HIV-1 reverse transcriptase inhibitory effect (20). However, there are no experimental data on anti-obesity effect of Rv-PEM01-99 from *R. verniciflua* leaves in mice.

In this paper, we demonstrated the anti-obesity effects of the Rv-PEM01-99 in high-fat diet (HFD)-induced obese mice to obtain new information of *R. verniciflua* leaves on general health as initial study.

## Materials and methods

### Preparation of Rv-PEM01-99

*R*. *verniciflua* leaves were collected from Wonju, Korea. Rv-PEM01-99 was prepared using a method described by Hiruma et al. (17). In brief, the powder of *R*. *verniciflua* leaves was extracted with 10 times the volume of 70% ethanol at 23–27°C, and the extracted solution was filtered, evaporated, and lyophilized. Using HPLC, we confirmed that Rv-PEM01-99 did not contain urushiol or its derivatives that cause skin allergy.

### Measurement of body weight, dietary intake, and organ weight in HFD-induced obese mice

The HFD-60 was purchased from Oriental Yeast Co., Ltd. (Tokyo, Japan). Six-week-old male C57BL/6J mice (Japan SLC, Inc., Shizuoka, Japan) were housed under a 12-h light/dark cycle at 23 ± 3°C. The mice were divided into three groups: group I (HFD group), group II (HFD-1% Rv-PEM01-99 group), and group III (HFD-2% Rv-PEM01-99 group). Composition of the experimental diets in this study is showed in Table 1. These mice were fed the respective diet for 56 days. Body weight and food intake were recorded twice a week. After 56 days of administration, blood biochemical analyses were carried out. Animal experiments were carried out at Japan Food Research Laboratories and were authorized by the Japanese Government, and the present study was conducted according to the ethical guidelines for laboratory animals and the standard operating procedure of the laboratory. The experimental protocol was approved by the animal experiment ethics committee of the laboratory (approval no. TM17072801).

**Table 1:** Composition of the experimental diets

Ingredient (g/kg)	Group		
	Group I	Group II	Group III
Casein	256.0	253.4	250.9
L-Cystine	3.6	3.6	3.5
Dextrose	60.0	59.4	58.8
Corn starch	160.0	158.4	156.8
Sucrose	55.0	54.5	53.9
Soybean oil	20.0	19.8	19.6
Lard	330.0	326.7	323.4
Cellulose	66.1	65.3	64.7
Mineral mixture	35.0	34.7	34.3
Calcium carbonate	1.8	1.8	1.8
Vitamin mixture	10.0	9.9	9.8
Choline bitartrate	2.5	2.5	2.5
Rv-PEM01-99	0.0	10.0	20.0
Total	1000.0	1000.0	1000.0

### Histological analysis in HFD-induced obese mice

The liver and mesentery adipose tissue from mice of each groups were fixed in 10% buffered formalin, embedded in paraffin. Some sections were stained with hematoxylin and eosin (H.E.) for histological examination and images were obtained using optical microscope.

### HPLC analysis

Rv-PEM01-99 extract was analyzed by HPLC using Agilent 1100 series HPLC system (Agilent Technologies Japan Ltd., Tokyo, Japan) equipped with a DAD G1315B photodiode array detector and TSKgel ODS-80Ts (4.6 i.d.× 250 mm) HPLC column (Tosoh, Tokyo, Japan). The mobile phase consisted of a linear gradient of 10 mM phosphoric acid–acetonitrile (95:5→5:95, 60 min), and flow rate was set at 0.8 mL/min (21). Measurement of urushiols was performed using the method of Du et al. (22) with some modification. The urushiols were measured using Develosil ODS-5 (10 i.d. × 250 mm) column (Nomura Chemical, Aichi, Japan) and Shimadzu LC6A system (Kyoto, Japan) equipped with an SPD-6AV UV-visible detector. The mobile phase consisted of 90% acetonitrile, and the flow rate was 1 mL/min with UV detection at 272 nm.

### LC/MS analysis

LC analysis was performed using Waters ACQUITY UPLC PLUS (Waters MS Technologies, Manchester, UK), which was equipped with a reversed-phase Acquity UPLC HSS T3 column with particle size 2.1 mm × 100 mm × 1.8 μm (Waters MS Technologies). The column oven temperature was set at 40°C and the flow rate was 0.4 mL/min. Mobile phases A and B consisted of water containing 0.1% formic acid and acetonitrile, respectively. The linear gradient program was set as follows: 0 min, 0.5% B; 21 min, 40% B; and 23 min, 100% B. The injection volume was 5 μL. Mass spectrometry was performed on a Xevo Qtof Mass Spectrometer (Waters MS Technologies). The scan range covered *m*/*z* from 50 to 1,000. For positive electrospray modes, the capillary and cone voltage were set at 3.0 kV and 15 V, respectively.

### Statistical analyses

The values are expressed as mean ± standard error (SE). Statistical significance was determined by paired *t*-test. The significance level was set at *P* < 0.05.

## Results

### Effects of Rv-PEM01-99 on body weight of HFD-fed C57BL/6J mice

**Fig. 1:**
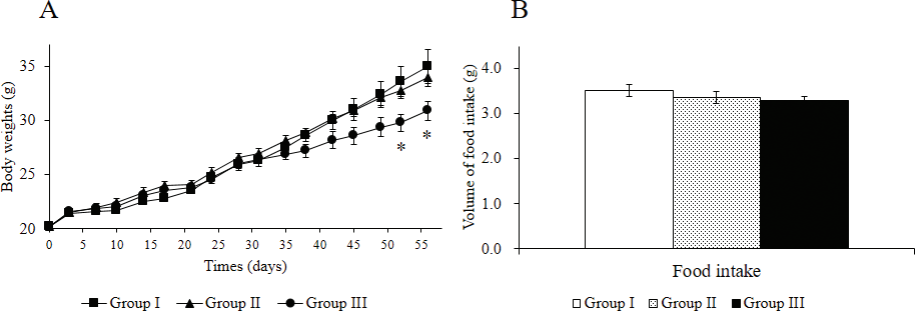
Effects of Rv-PEM01-99 on body weight of C57BL/6J mice after 56 days of administration. (A) Body weights (g) and (B) food intake (g) in Group I (HFD group), Group II (HFD-1% Rv-PEM01-99 group), and Group III (HFD-2% Rv-PEM01-99 group). Results are expressed as mean ± S.E. (*n* = 6).*P < 0.05 vs. Group I.

The effects of oral administration of Rv-PEM01-99 on body weight and food intake of HFD-fed C57BL/6J mice HFD are shown in Fig. 1. No significant differences were observed in food intake volume between groups I, II, and III (Fig. 1B). Conversely, oral administration of the Rv-PEM01-99 suppressed body weight gain in mice as compared to group I. In particular, group III showed significantly reduced body weight of mice compared to group I after 52 days of administration, and group III treatment caused a decrease in the body weight by approximately 11% as compared to group I at 52 and 56 days, respectively (Fig. 1A).

### Effects of Rv-PEM01-99 on organ weight and intra-abdominal fat of C57BL/6J mice

**Fig. 2:**
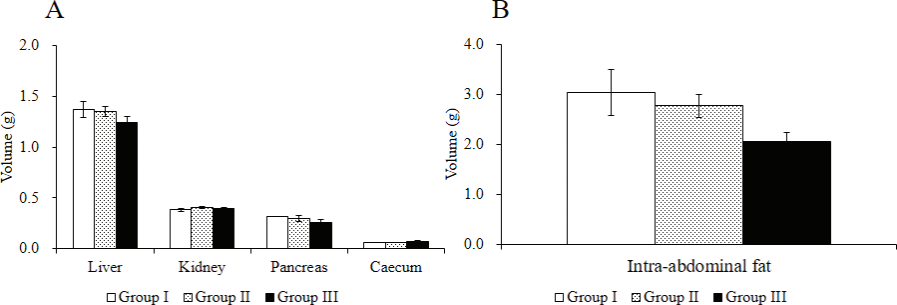
Effects of Rv-PEM01-99 on organ weight in C57BL/6J mice after 56 days of administration. (A) Organ weight (g) and (B) intra-abdominal fat level (g) in Group I (HFD group), Group II (HFD-1% Rv-PEM01-99 group), and Group III (HFD-2% Rv-PEM01-99 group). Results are expressed as mean ± S.E. (*n* = 6).

The results of investigation of organ weight and intra-abdominal fat in mice after administration of Rv-PEM01-99 for 56 days are shown in Fig. 2. No significant differences were obtained in volume of liver, kidney, pancreas, and caecum between groups I, II, and III (Fig. 2A). The intra-abdominal fat levels (mesentery, circumference of kidney, circumference of testicle) of mice in group III were lower than those of group I after oral administration of Rv-PEM01-99 for 56 days (Fig. 2B).

### Effects of Rv-PEM01-99 on serum biochemical parameters of C57BL/6J mice

**Fig. 3:**
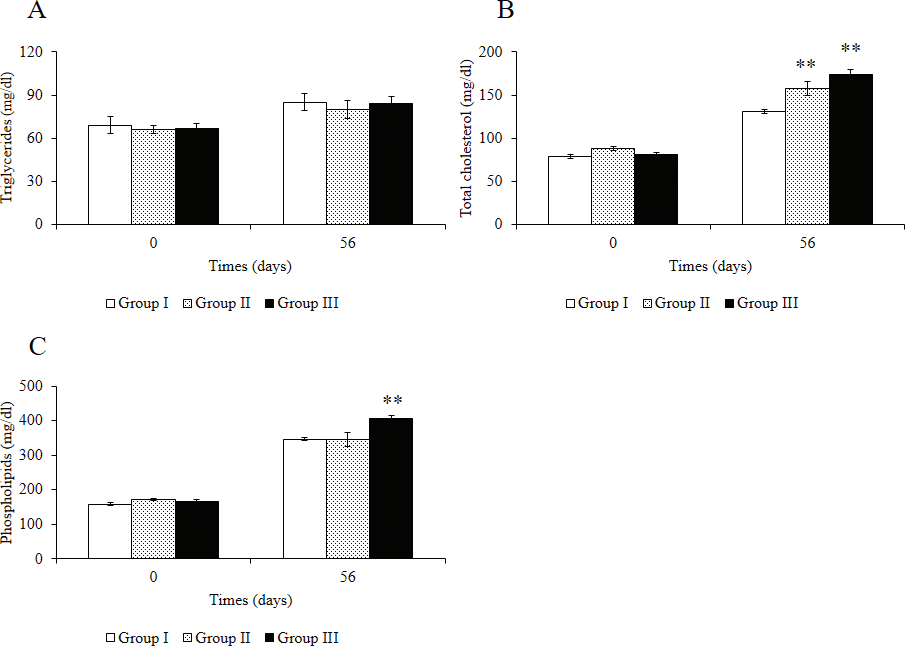
Effects of Rv-PEM01-99 on plasma obesity biomarker levels in C57BL/6J mice after 56 days of administration. (A) TG (mg/dL), (B) TC (mg/dL), (C) PL (mg/dL). Group I (HFD group), Group II (HFD-1% Rv-PEM01-99 group), and Group III (HFD-2% Rv-PEM01-99 group). Results are expressed as mean ± S.E. (*n* = 6).**P < 0.01 vs Group I.

**Fig. 4:**
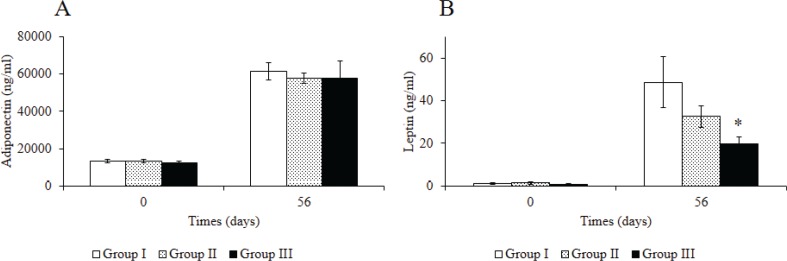
Effects of Rv-PEM01-99 on plasma obesity biomarker levels in C57BL/6J mice after 56 days of administration. (A) Adiponectin (ng/mL) and (B) leptin (ng/mL) levels in Group I (HFD group), Group II (HFD-1% Rv-PEM01-99 group), and Group III (HFD-2% Rv-PEM01-99 group). Results are expressed as mean ± S.E. (*n* = 6).*P < 0.05 vs. Group I.

Figures 3 and 4 show the analysis of biochemical parameters of mice after administration of Rv-PEM01-99. No marked differences were seen in triglyceride (TG) concentrations between groups I, II, and III after 56 days of Rv-PEM01-99 administration (Fig. 3A). On the contrary, the total cholesterol (TC) levels in groups II and III were significantly higher than those in group I after 56 days (Fig. 3B), and the phospholipid (PL) levels in group III were significantly higher than those in group I after 56 days (Fig. 3C). Leptin level in group III was lower than that in group I (Fig. 4B).

### Effects of Rv-PEM01-99 on liver and mesentery adipose tissue of C57BL/6J mice

**Fig. 5:**
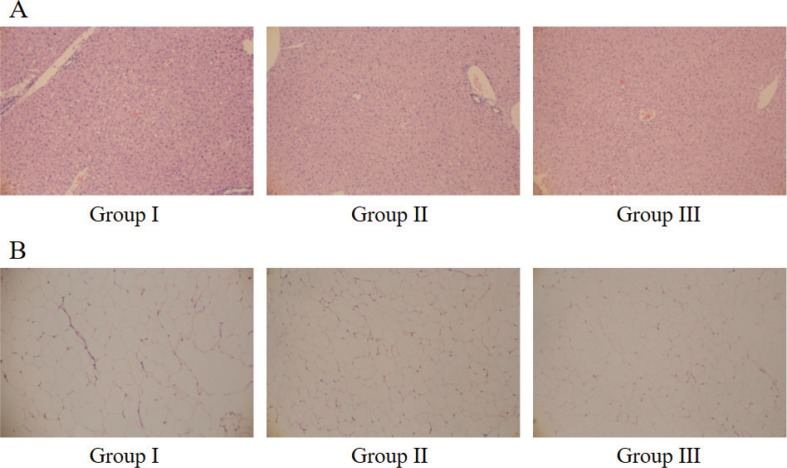
Histological analysis in HFD-induced obese mice. (A) liver tissue, (B) mesentery adipose tissue. The liver and mesentery adipose tissue from mice of each groups were fixed in 10% buffered formalin, embedded in paraffin. Some sections were stained with H.E. for histological examination and images were obtained using optical microscope.

The images of liver and mesentery adipose tissue from mice of each groups are shown in Fig. 5. No significant differences were observed in tissue analysis between groups I, II, and III.

### HPLC and LC/MS analysis of Rv-PEM01-99

**Fig. 6:**
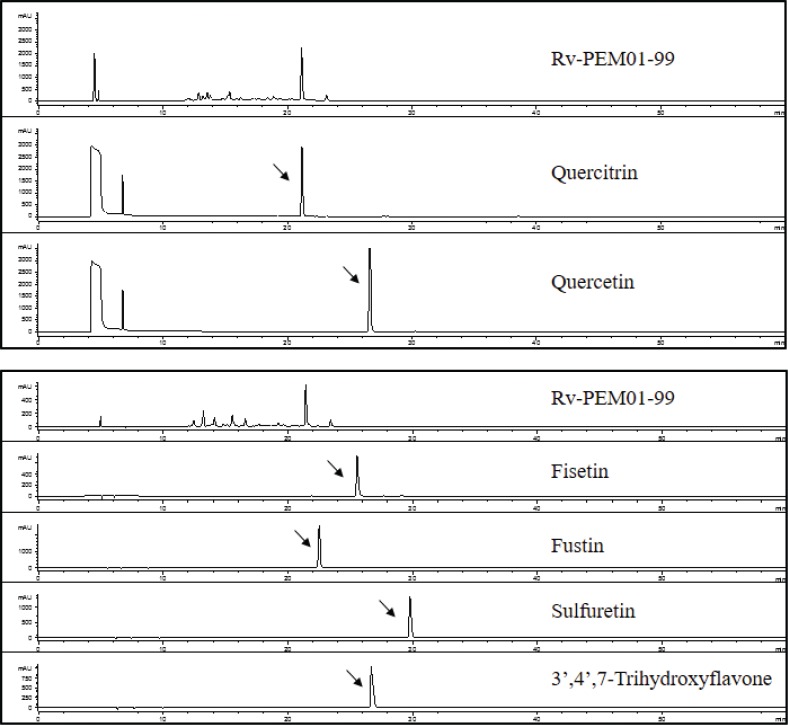
HPLC profile of Rv-PEM01-99.

**Fig. 7:**
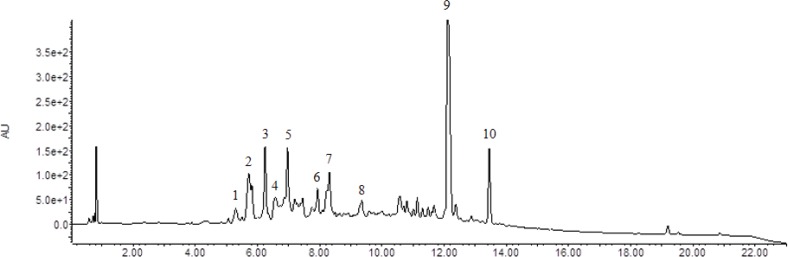
LC/MS profile of Rv-PEM01-99.

Few urushiols and their derivatives were detected in Rv-PEM01-99 by HPLC analysis (data not shown). The HPLC chromatogram of Rv-PEM01-99 is shown in Fig. 6. Rv-PEM01-99 contained quercitrin in the form of a quercetin derivative, but it did not contain quercetin, fisetin, fustin, sulfuretin, and 3’,4’,7-trihydroxyflavone. The chemical profiles of Rv-PEM01-99 were analyzed using LC/MS (Fig. 7). The 10 compounds identified were detected in Rv-PEM01-99, and 5 out of the 10 compounds were suggested based on retention time, MS data (Table 2).

**Table 2:** LC/MS data of compounds identified in the Rv-PEM01-99

Peak No.	Retention time (min)	MS (m/z)	Molecular formula	Suggested compound
1	5.376	299.0790	C13H14O8	Unidentified
2	5.784	299.0818	C13H14O8	Unidentified
3	6.319	299.0802	C13H14O8	Unidentified
4	6.628	355.0969	C16H18O9	Chlorogenic acid
5	7.025	355.0915	C16H18O9	Chlorogenic acid
6	7.997	299.0800	C13H14O8	Unidentified
7	8.376	291.0789		Unidentified
8	9.419	339.1055	C12H18O11	Glucopyranosyl-L-ascorbic acid
9	12.207	449.0884	C21H20O11	Quercitrin
10	13.533	433.0962	C21H20O10	Afzelin

## Discussion

Increased body weight and obesity are rapidly becoming a common occurrence due to busy lifestyles all over the world, especially in developed countries. High body weight and obesity drive many serious diseases, such as high blood pressure, diabetes, and cancer (3). Generally, obesity and high body weight can be avoided by following calorie restriction, sugar restriction, and exercise programs (6). However, following these solutions can be difficult for obese people. Clinically available anti-obesity agents and/or drugs include orlistat (23), lorcaserin (24), norepinephrine (25), isoproterenol (26), and phoscholine (27). However, these anti-obesity agents show severe side effects, such as constipation, insomnia, vomiting, headache, abdominal pain, and myocardial infarction (28), and therefore, have limited applications. Hence, there is a need for development of anti-obesity agents with minimal adverse effects from natural sources like plants and herbs.

Research on phytochemicals from fruits, vegetables, mushrooms, and herbs has focused on their potential benefits. Some of these have been used as folk medicines, sources of physiologically beneficial medicines, and raw materials for functional foods. Phenolic compounds are important natural antioxidants and show inhibitory activity against carbohydrate hydrolyzing enzymes (29). Many researchers have evaluated the antifibrogenic, antimutagenic, antioxidant, and antitumorigenic activities of phenolic compounds from *R. verniciflua* bark, heartwood, and branch (30). Moreover, Kim et al. demonstrated that *R. verniciflua* heartwood and stem extracts showed a strong alpha-glucosidase inhibitory activity (13, 31). However, there are no experimental reports on the anti-obesity effects of *R. verniciflua*. In this study, Rv-PEM01-99 was prepared from *R. verniciflua* leaves and its anti-obesity effects in HFD-induced obese mice were determined to develop a new functional food material and/or nutritional supplement for human healthcare as initial study. The doses of Rv-PEM01-99 in this mice study were installed using a method described by Miyata et al. (32) because they reported the anti-obesity effect of the aqueous extract from *Houttuynia cordata*Thunb leaves containing quercitrin.

No deaths or abnormalities in food consumption and coat condition were noted in the Rv-PEM01-99-treated mice in this study. We confirmed that Rv-PEM01-99 did not contain urushiol or its derivatives that cause skin allergy on HPLC analysis. In previous paper, we tried the acute oral toxicity study of the extract from *R. verniciflua* leaves in mice, and reported that it did not identify any adverse reaction compared to control group (17). Moreover, we investigated the effect of Rv-PEM01-99 on plasma concentration of metabolites in obese dogs for 6 weeks administration, and confirmed that the Rv-PEM01-99 did not show any adverse reaction compared to control group (33). From these results, we believe that Rv-PEM01-99 do not show side effects in mice and dogs; however, we consider that it is important to have more detail toxicity data such as liver histology, and this may be one of next study on Rv-PEM01-99.

Oral administration of the Rv-PEM01-99 suppressed body weight gain, and group III showed greater reduction in body weight than group I after 52 days of administration. In this study, no marked weight loss in the group II mice was observed when compared to group I upon 56 days administration, but we expect that group II may have the similar effects to group III, resulting in suppression of body weight gain during longer study protocols such as 84, 112, or 140 days of Rv-PEM01-99 administration. The intra-abdominal fat levels of mice in group III were lower than those in group I after oral administration for 56 days. Moreover, group III treatment suppressed the increase in leptin concentration as compared to group I. Leptin is a key adipose-derived regulator of energy expenditure and food intake (34). Patients with leptin deficiency show excess appetite and obesity (35). However, in general population, high levels of leptin cannot decrease body weight (36). A recent report suggests that the leptin levels showed a significant correlation with the area of intra-abdominal fat (37). In line with previous studies, our data indicate that anti-obesity effects of Rv-PEM01-99 are related to the suppression of intra-abdominal fat and leptin levels. On the contrary, the TC and PL levels in group III were significantly higher than those in group I after 56 days. In our previous studies, we investigated the effect of Rv-PEM01-99 on plasma concentration of metabolites in obese dogs, and Rv-PEM01-99 showed a potent antioxidant effect on malondialdehyde levels, and a marked improvement in liver function (as measured by lactate dehydrogenase, LDH activity) (33). From these results, we speculate that the TC and PL levels of mice in group III were significantly higher than that of group I because of no deterioration in liver function by administration of Rv-PEM01-99 compared to group I. This study is a preliminary investigation about anti-obesity effects of Rv-PEM01-99 on HFD-fed mice. So, the mechanisms of Rv-PEM01-99 on anti-obesity effects remain unclear. Therefore, we have to obtain more data about effects of Rv-PEM01-99 on adipogenesis, thermogenesis, insulin sensitivity, detail adipose histology, and other biomarkers and so on. Moreover, we need the compare between Rv-PEM01-99 and orlistat, lorcaserin, nerepinephrine, and things like that as positive control.

HPLC analysis shows that Rv-PEM01-99 contains quercitrin, aquercetin derivative, as one of the active compounds. Some investigators reported the antioxidant activity of the flavonoid quercetin containing quercitrin (38–40). Moreover, Miyata et al. investigated the anti-obesity effect of the aqueous extract from *H. cordata* Thunb leaves containing quercitrin. They showed that this extract exerted anti-obesity effects by inhibiting nonesterified fatty acid and glycerol absorption from the small intestine in mice (32). The anti-obesity effects of Rv-PEM01-99 may be related to quercitrin. In addition to quercitrin, chlorogenic acid, glucopyranosyl-L-ascorbic acid, and afzelin might be active components of Rv-PEM01-99, and are yet to be identified and validated.

## Conclusion

In summary, the anti-obesity effects of Rv-PEM01-99 extract from *R. verniciflua* leaves were shown. Our results show that Rv-PEM01-99 contains a quercetin derivative, which showed an anti-obesity effect on HFD-fed mice. Rv-PEM01-99 did not contain urushiol or its derivatives that act as allergic components, and no deaths or abnormalities in food consumption and coat condition were observed in Rv-PEM01-99-treated mice. The underlying mechanisms of Rv-PEM01-99 on anti-obesity effects remain unclear; however, it could be used as anedible component and/or nutritional supplement for management of obesity.
